# A screening protocol for child abuse at out-of-hours primary care locations: a descriptive study

**DOI:** 10.1186/s12875-016-0554-4

**Published:** 2016-11-08

**Authors:** Maartje C. M. Schouten, Henk F. van Stel, Theo J. M. Verheij, Edward E. S. Nieuwenhuis, Elise M. van de Putte

**Affiliations:** 1 Wilhelmina Children’s Hospital, University Medical Center, Utrecht, The Netherlands; 2 Julius Center for Health Sciences and Primary Care, University Medical Center, Utrecht, The Netherlands; 3 Division of Pediatrics, Wilhelmina Children’s Hospital, Postbox 85090, 3508 AB Utrecht, The Netherlands

**Keywords:** Child abuse, Mass screening, Primary health care

## Abstract

**Background:**

Child abuse is often unrecognized at out-of-hours primary care (OOH-PC) services. The aim of our study was to evaluate the clinical outcome of the screening instrument SPUTOVAMO-R2 for child abuse (checklist), followed by a structured approach (reporting code), at OOH-PC services. The reporting code with five steps should ensure consistent action in case of a suspicion.

**Methods:**

All children attending one of the five participating OOH-PC services in the region of Utrecht, the Netherlands, in a year time, were included. The checklist is an obligatory field in the electronic patient file and was filled in for all children. In case of a positive checklist, the steps in the reporting code were followed. Additionally, the case was evaluated in a multidisciplinary team to determine the probability of child abuse.

**Results:**

The checklist was filled in for 50671 children; 108 (0.2 %) were positive. The multidisciplinary team diagnosed child abuse in 24 (22 %) of the 108 positive checklists, and no child abuse in 36 (33 %). Emotional neglect was the most frequent type of abuse diagnosed. For all abused children, care was implemented according to the protocol. The most frequent care given was a referral to the hospital (*N* = 7) or contact with child’s own general practitioner (*N* = 6).

**Conclusion:**

A checklist followed by a reporting code guarantees consistent actions and care for children with a suspicion of child abuse. The percentage of positive checklists is lower than expected. Validity of the checklist should be assessed in a diagnostic study.

**Electronic supplementary material:**

The online version of this article (doi:10.1186/s12875-016-0554-4) contains supplementary material, which is available to authorized users.

## Background

Child abuse can have major negative consequences for the child, family and society. Child abuse is estimated to have a prevalence of 34 in 1000 children, in the Netherlands in 2010 [1]. Child maltreatment is defined as any act or series of acts of commission or omission by a parent or other caregiver that results in harm, potential for harm, or threat of harm to a child [2]. Child abuse is not only inflicted physical injury resulting in bruises, fractures, burns et cetera. It also includes emotional abuse and neglect such as emotional unavailability, negative attributions and inappropriate developmental expectations. Sexual abuse and factitious disorders are more infrequent manifestations of child abuse. With consequences such as serious long-term medical and mental health problems, early detection of child abuse is essential [3–5].

**Fig. 1:**
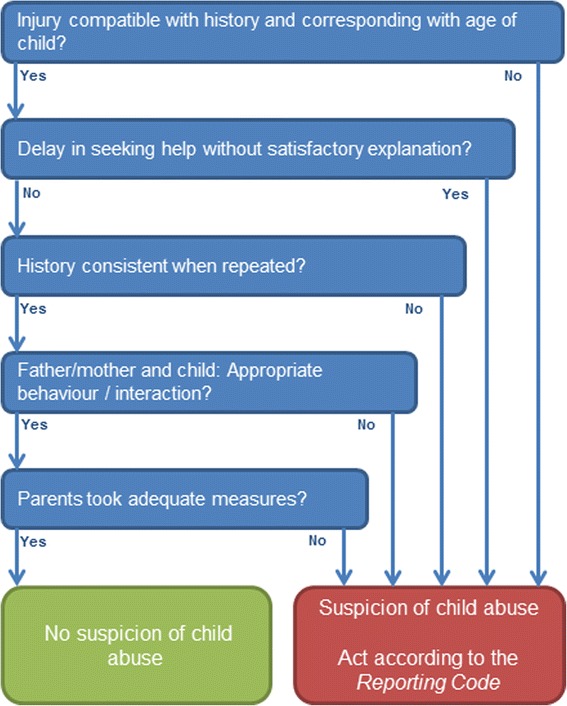
SPUTOVAMO-R2 screening instrument for child abuse

**Fig. 2:**
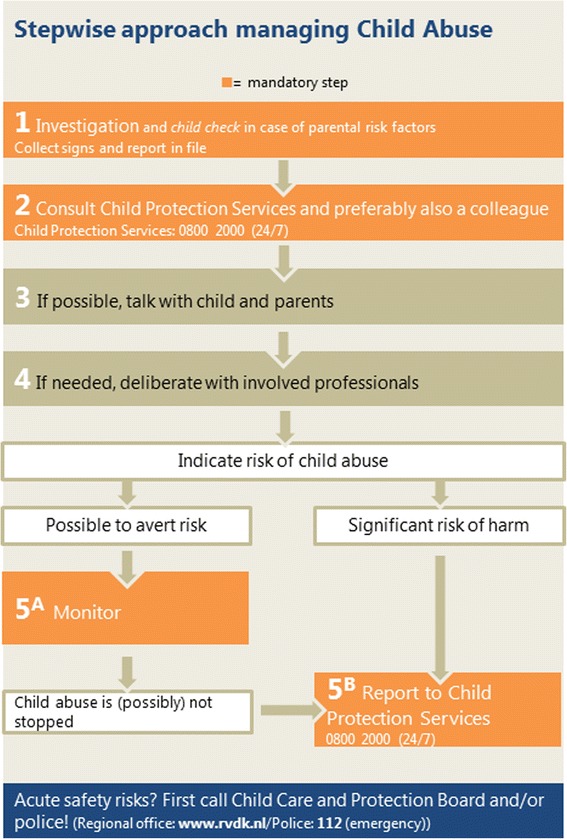
KNMG-Reporting Code for medical doctors in case of a suspicion of child abuse (translation of the Dutch KNMG-meldcode [14]; translated in cooperation with the KNMG)

Out-of–hours primary care (OOH-PC) services are an important place for detecting child abuse. Firstly, children are over presented in the OOH-PC compared to the family day practice [6]. Secondly, child and family are relatively anonymous at an OOH-PC service. Lastly, at the moment of presentation, parents are in need of care for their child, and this might create a window-of-opportunity to have a dialogue about safety concerns [7]. Nevertheless, child abuse is often unrecognized in the OOH-PC setting [8]. Screening instruments can assist health professionals in their awareness of child abuse, leading to an increased detection [9–11]. Therefore, the Dutch Inspection for Health Care stated in 2010, that all OOH-PC services are obliged to have a reliable screening instrument for child abuse [12]. One of the tools for screening for child abuse is the widely used screening instrument SPUTOVAMO. Originally designed as screening tool for emergency rooms, it can also be used in the OOH-PC [12]. The original SPUTOVAMO consists of nine open questions directed at the injury, development stage of the child and actions of parents. SPUTOVAMO was revised into a checklist with six questions (SPUTOVAMO-R), which resembles the detection instrument of Benger et al [13]. The binary answer possibilities of this SPUTOVAMO-R primarily point at the suspicion of physical child abuse or not. SPUTOVAMO-R was revised into SPUTOVAMO-R2 for the OOH-PC. SPUTOVAMO-R2 consists of five questions, directed not only at the injury, but also at the interaction with parents and child. These five questions support the health professional to consider each type of child abuse. For example, if the child is completely ignored during the OOH-PC visit or no affection is shown by parents when the child is clearly in need of comfort, the interaction is not appropriate (i.e. suspicion of emotional neglect). The screening instrument is short on purpose, to make use in the busy OOH-PC feasible. With one deviant answer, the screening instrument classifies positive for the suspicion of child abuse (Fig. 1). In case of a suspicion of child abuse, medical doctors are mandated to act according to the five steps of the reporting code for child abuse (*KNMG-reporting code*) (Fig. 2) [14, 15]. This reporting code ensures a thorough diagnostic process and careful communication with patient and family. With consistent use of the steps, a sound decision on whether to report to child protection services (CPS) can be reached [15]. In the Netherlands, medical doctors have the right, but not the obligation, to report to CPS [16, 17]. Depending on the willingness and potentials of parents to avert safety risks, medical doctors have the possibility to either implement care and monitor the situation, or to report the child to CPS.

Comprehensive evidence on the accuracy of screening instruments for child abuse is limited [10, 18]. Also, the effect of the reporting code in case of a suspicion has not yet been studied, and it is unclear if the reporting code ensures consistent actions and care for the child. With mandatory screening protocols implemented nationwide at OOH-PC services in the Netherlands, more investment in, and knowledge about, a structured approach after positive screening is needed [9].

In this study, we aimed to assess the clinical outcome of the screening instrument SPUTOVAMO-R2 for child abuse followed by a structured approach (reporting code), at OOH-PC services. The outcome was defined as a multidisciplinary assessment of child abuse of the children with a positive screening.

## Methods

### Clinical procedure

All the five participating OOH-PC services around the city of Utrecht, the Netherlands, are unified in one organization (*Primair Huisartsenposten*). This organization facilitates out-of-hours primary care with nine locations in a catchment area of around 1.5 million people. Around 265.600 children 0-18 years where living in this region in the study year. In 2012, there were 232.187 patient contacts in five OOH-PC services [19]. The prevalence of child abuse reported to child protection services for the study region was 0.8 % in 2012 (Netherlands Youth Institute, information on request, 2015). The SPUTOVAMO-R2 – to which we will further refer as the checklist - is filled in for all patients under the age of 18. This is an obligatory field in the electronic patient file at the OOH-PC service. All general practitioners (GP) working at the OOH-PC services are familiar with the reporting code (Fig. 2).

For all the children contacting the OOH-PC between July 2012 and July 2013, the GP assessed the possibility of child abuse with the help of the checklist. In case of a positive checklist, more questions concerning parental risk factors and a top-toe examination followed (screening protocol; Additional file 1). Depending on the outcome of the checklist (i.e. positive or negative), the steps in the reporting code (Fig. 2) were followed and the case was evaluated in a multidisciplinary team.

All the GPs working at the OOH-PC services were offered a training for recognizing child abuse using an e-learning program ‘The Next Page’ and communication training [20].

### Multidisciplinary team

The multidisciplinary team consisted of the involved GP(s) of the child; a ‘child abuse professional’ of the OOH-PC service; a CPS doctor; and a ‘child abuse paediatrician’ of the University Medical Center Utrecht. The multidisciplinary team discussed all children with a positive checklist result in monthly meetings. Before the meeting, extra information was gathered from the GP that scored the checklist positive in the OOH-PC, their own GP and child services, to make a risk assessment of the child and the family. In the meetings, the following information of each child was presented in a structured way: filled-in checklist, answers on the extra questions asked in the OOH-PC, and the risk assessments (e.g. information concerning if the child was already known at child services (CPS and/or Youth Care)). Youth Care focusses on raising families in a safe environment. A child can be referred to Youth Care for different reasons, not exclusively child abuse, and a referral to Youth Care is always voluntary. Based on the given information, the multidisciplinary team answered three main questions: 1) the probability of child abuse, 2) in case of child abuse, which type of abuse, and 3) the indicated care. The diagnosis of child abuse was defined as a consensus agreement on child abuse by the multidisciplinary team. If (specific) care was needed and not yet implemented, it was started after the meeting by the child’s own GP.

### Ethical approval

Children and their parents were informed about the extra care for child safety through posters and information flyers at the OOH-PC services; the flyers were personally given to them. All children received clinical care according to the screening protocol (*Primair Huisartsenposten;* Additional file 1 *)* and national guidelines (reporting code), independently of this study [14]. Evaluation of the children with a positive checklist within the multidisciplinary team is standard care in the OOH-PC. The researcher received all data anonymous. According to the Dutch Medical Research Involving Human Subjects Act, this kind of observational study is exempt from ethical review (confirmed by the Medical Ethical Commission UMC Utrecht, protocol number 12–286/C).

### Statistical analyses

Characteristics of the children and outcomes of the multidisciplinary team were described using means and percentages. Risk factors were analysed as odds ratios for child abuse (with 95 % confidence interval). Differences were considered significant if *p* was less than 0.05. All analyses were performed using SPSS version 21.

## Results

### Outcomes of the multidisciplinary team

The checklist was filled in for all the 50671 children who attended one of the five participating OOH-PC services (100 % screening rate). Of these, 108 (0.2 %) had a positive checklist. The median age at the moment of OOH-PC contact was 3.0 years (IQR 1.0–8.0); 46 % was male.

The multidisciplinary team concluded that there was child abuse in 24 (22 %) of the 108 screen positive children; in 28 (26 %) there was no conclusion because suspicion of child abuse was already discarded (*n* = 26), or because the child’s own GP did not concur with multidisciplinary deliberation. No child abuse was concluded in 36 (33 %) children. Twenty children (19 %) were not diagnosed by the multidisciplinary team because of missing information and absence of the involved GPs (Table 1).

**Table 1:** Demographics of screen positive children, according to conclusion of the multidisciplinary team on child abuse

Conclusion multidisciplinary team	Child abuse cases (*n* = 24)		No child abuse cases (*n* = 36)		Suspicion discarded before meeting (*n* = 28)		No conclusion due to missing information (*n* = 20)	
	N	%	N	%	N	%	N	%
Gender								
Girl	12	50	22	61	14	50	10	50
Age								
≤ 4 years	12	50	24	67	18	64	9	45
5–11 years	9	38	6	17	6	21	5	25
12–18 years	3	12	6	17	4	14	6	30
Child known to child services								
yes	16	67	12	33	-^a^	-^a^	1^a^	5
no	8	33	24	67	3	11	1^a^	5

^a^Missing information

**Fig. 3:**
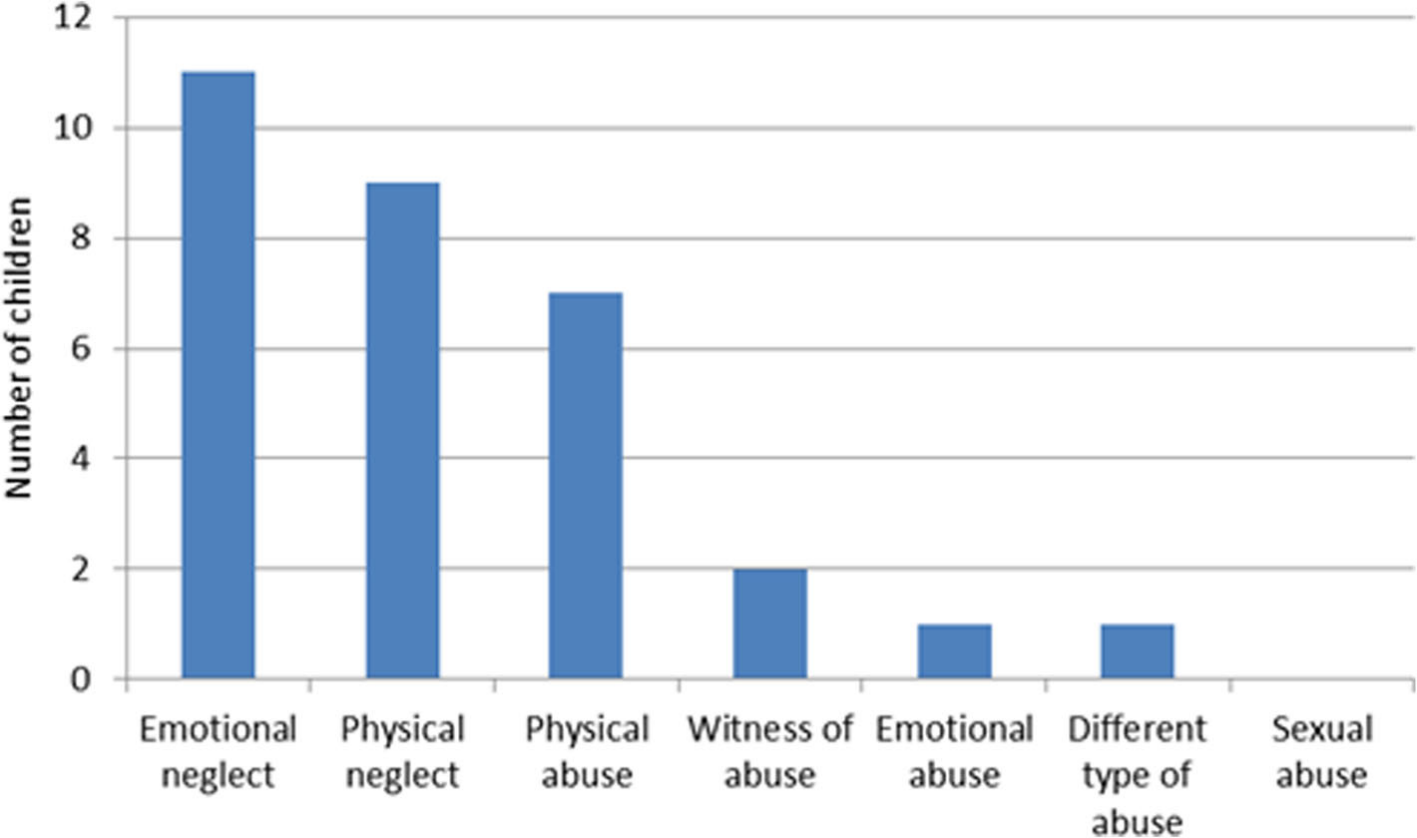
Type of child abuse in 24 children (6 children with multiple types of abuse) as diagnosed by the multidisciplinary team out of 108 screened positives

Of the children diagnosed as abused, emotional neglect was the most frequent type of abuse (*N* = 11), followed by physical neglect (*N* = 9) (Fig. 3). Question 4 was most frequently answered deviant on the checklist (*n* = 17) (Table 2). A total of 28 children were already known at child services (CPS and/or Youth Care) before the OOH-PC visit: 16 in the group of 24 children diagnosed with child abuse, and 12 in the group of 36 children diagnosed with no child abuse. Children already known at child services, had a significantly higher odds for child abuse than the children unknown (OR 4.00, 95 % CI 1.34–11.96, *p* = 0.013).

**Table 2:** Questions answered deviant on the SPUTOVAMO-R2 checklist for each type of child abuse^a^

	Question deviant SPUTOVAMO-R2	Q1	Q2	Q3	Q4	Q5
Type of abuse						
Emotional neglect (*n* = 11)		1	5	1	7	6
Physical neglect (*n* = 9)		1	4	0	4	4
Physical abuse (*n* = 7)		4	2	1	2	3
Witness of abuse (*n* = 2)		0	1	0	2	1
Emotional abuse (*n* = 1)		0	0	0	1	1
Different type of abuse (*n* = 1)		0	0	1	1	0
Sexual abuse (*n* = 0)		0	0	0	0	0
Total		6	12	3	17	15

Q1: Injury compatible with history and corresponding with age of child?

Q2: Delay in seeking help without satisfactory explanation?

Q3: History consistent when repeated?

Q4: Father/mother and child: appropriate behaviour/interaction?

Q5: Parents took adequate measures?

^a^Multiple deviant answers possible in one case

### Care implemented

All of the children diagnosed with child abuse by the multidisciplinary team, received care. In five children, no explanation was given about the specific care implemented. Of the other 19 children, six children received multiple forms of care. The most frequent care given at the OOH-PC services was a referral to the hospital (emergency room or paediatrician) (*N* = 7) or contact with child’s own GP (*N* = 6). Other actions were: reporting the child to Youth Care (*N* = 4) or reporting the child to CPS (*N* = 1), an appointment with the child’s own GP the next week (*N* = 3), and isolating the child from the perpetrator (*N* = 3). Isolating the child from the perpetrator was the care given for children with physical abuse or physical neglect. Reporting to CPS was done in one case of physical and emotional neglect. A referral to the hospital was the care given for children with physical abuse/neglect or emotional abuse/neglect.

## Discussion

To our knowledge, this is the first article describing a checklist for child abuse followed by a structured approach (reporting code) at OOH-PC services. With a structured approach, the ultimate goal is to guarantee the safety of each child attending the OOH-PC. Since it is mandatory, all the 50671 children attending the OOH-PC were screened for child abuse with the checklist. Of the 108 children with a positive checklist, the multidisciplinary team could confirm child abuse in 24 children. For those 24 children, care was implemented.

Some limitations need to be discussed. Firstly, the evaluation by the multidisciplinary group was only applied to the children with a positive checklist. Therefore, it was impossible to determine the validity of the checklist. Secondly, not all children with a positive checklist were evaluated by the multidisciplinary team because of missing information. Thirdly, due to lack of follow up and evaluation of the care, it was impossible to draw conclusions about the appropriateness of the care for the abused children. However, this is the first study to evaluate the mandatory reporting code with a high rank reference test (multidisciplinary team assessment).

To justify the use of a screening instrument, it is important to – at least - establish the diagnostic value. At this moment, the screening protocol includes the screening instrument SPUTOVAMO-R2, which lacks validation. The diagnostic value of SPUTOVAMO-R is assessed at emergency rooms, and showed a low positive predictive value (3 %) for inflicted injury [21]. In our study, with only the children with a positive checklist deliberated in the multidisciplinary team, it is impossible to know if cases have been missed. In addition, not all positive screens were true positives (in 33 % there was no child abuse). With a low prevalence of positive checklists (0.2 %), one could argue about the cost-effectiveness ratio of mandatory use of a screening instrument. Other studies determined a prevalence of positive checklists of 1.6 to 2.6 %, with confirmation of child abuse in 3 to 69 % of those positive checklists [7, 10, 21, 22]. We do not have a valid explanation for the difference in prevalence of positive checklists at OOH-PC services (0.2 %) compared to the prevalence of positive checklists at emergency rooms (1.6–2.6 %). At an emergency room, more severe injuries might be presented than in the OOH-PC, but this is not necessarily an explanation for the assessed difference in prevalence. It might be that GPs are still unaware of the possibility of child abuse or do not recognize the symptoms [8]. A screening instrument increases the awareness of abuse [9–11]. No information is available on the detection rate of child abuse at OOH-PC services, and a comparison between before and after introduction of the checklist in the OOH-PC is therefore not possible.

The outcome of the multidisciplinary team was used as reference test, in our study. With the clinical information and known outcome of the checklist used in the multidisciplinary team, incorporation bias might have occurred, leading to an overestimation of child abuse cases. The finding of having a significantly higher odds for child abuse when the child is already known at child services could also be an overestimation; given that the information regarding previous report to CPS or Youth Care was used in the assessment of child abuse by the multidisciplinary team. An inventory among clinicians showed that children previously reported to child services are more likely to be reported to CPS of a suspicion [23].

Emotional neglect was the most frequent type of abuse found in our study, followed by physical neglect. Emotional neglect can be difficult to detect because it is impossible to recognize a persistent pattern in the OOH-PC (a one-time encounter). However, with the use of the checklist, also children who were a victim of emotional neglect were identified. In the checklist, especially the question on the interaction with parents and child was answered deviant; emphasizing that this is an important question for identifying children with emotional neglect (Table 2). Unfortunately, we have no information on repeated visits of the screened children, to assess if there is a pattern. Noteworthy, physical neglect is the 6th most found type of abuse in the Netherlands versus second most found in our study [24].

The use of the reporting code guarantees care for each individual child with a suspicion; for all these children actions were undertaken and care was implemented. Only one out of the 24 children with diagnosis of child abuse was reported to CPS by the GP at the OOH-PC service. This low frequency of reporting to CPS could be due to the other possibilities given in the reporting code to stop a situation of child abuse, and the fact that medical doctors have no obligation to report a suspicion of child abuse [16]. Because of privacy reasons, we could not assess whether the implemented care was on hindsight the most appropriate action for the individual child. Nevertheless, all the implemented care were possible actions given in the screening protocol of the OOH-PC services (Additional file 1).

## Conclusion

In conclusion, a checklist followed by a reporting code promotes structured actions and care for children with a suspicion of child abuse. The percentage of positive checklists is lower than expected. Further research on the validation of the screening instrument SPUTOVAMO-R2 for child abuse at OOH-PC services is needed. In addition, an assessment of the cost-effectiveness ratio in terms of the possibility to avert safety risks with this screening procedure, is warranted.

## Additional file

Additional file 1: Screening protocol child abuse, *Primair Huisartsenposten,* represented by a flow chart for the general practitioners (whole screening protocol consisting of 12 pages is available on request). (TIF 119 kb)

## Abbreviations

CPS: Child protection services
GP: General practitioner
OOH-PC: Out-of-hours primary care
