# Supplementary figures and images for: A screening protocol for child abuse at out-of-hours primary care locations: a descriptive study
Source: BMC Fam Pract. 2016 Nov 8;17:155. doi: 10.1186/s12875-016-0554-4 (PMC5101665; doi:10.1186/s12875-016-0554-4)

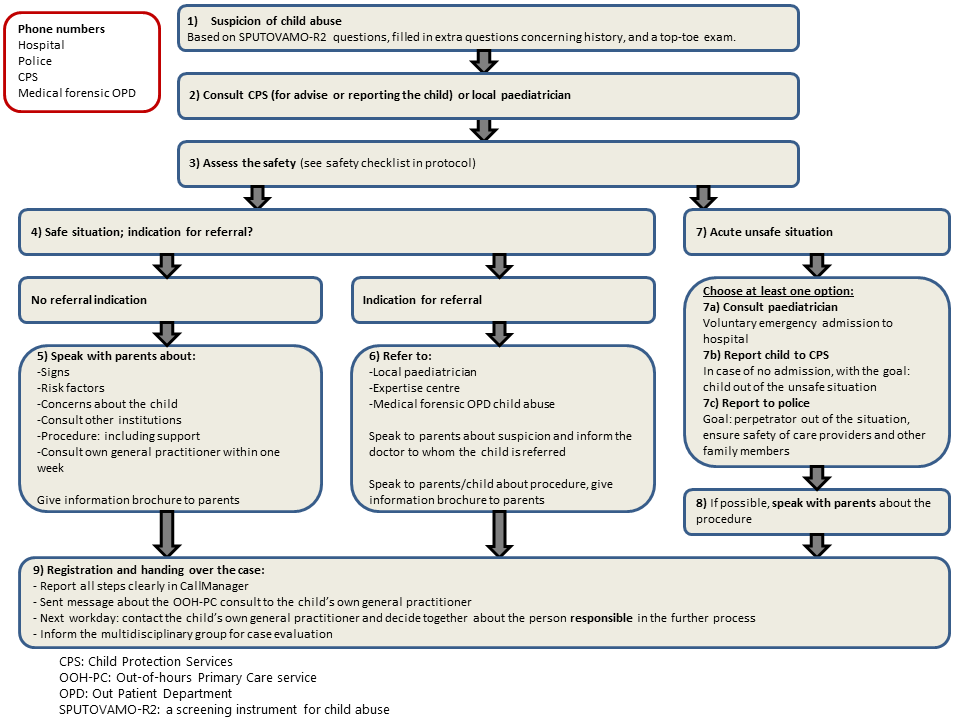

Supplement: Additional file 1: — Screening protocol child abuse, Primair Huisartsenposten, represented by a flow chart for the general practitioners (whole screening protocol consisting of 12 pages is available on request). (TIF 119 kb) [file 12875_2016_554_MOESM1_ESM.tif]
